# Evidence for Bladder Urothelial Pathophysiology in Functional Bladder Disorders

**DOI:** 10.1155/2014/865463

**Published:** 2014-05-08

**Authors:** Susan K. Keay, Lori A. Birder, Toby C. Chai

**Affiliations:** ^1^Division of Infectious Diseases, University of Maryland School of Medicine and the VA Maryland Health Care System, 10 North Greene Street, Room 3B-184, Baltimore, MD 21201, USA; ^2^Departments of Medicine and Pharmacology & Chemical Biology, University of Pittsburgh School of Medicine, A 1217 Scaife Hall, 3550 Terrace Street, Pittsburgh, PA 15261, USA; ^3^Department of Urology, Yale School of Medicine, 789 Howard Avenue, FMP 309, P.O. Box 208058, New Haven, CT 06519-8058, USA

## Abstract

Understanding of the role of urothelium in regulating bladder function is continuing to evolve. While the urothelium is thought to function primarily as a barrier for preventing injurious substances and microorganisms from gaining access to bladder stroma and upper urinary tract, studies indicate it may also function in cell signaling events relating to voiding function. This review highlights urothelial abnormalities in bladder pain syndrome/interstitial cystitis (BPS/IC), feline interstitial cystitis (FIC), and nonneurogenic idiopathic overactive bladder (OAB). These bladder conditions are typified by lower urinary tract symptoms including urinary frequency, urgency, urgency incontinence, nocturia, and bladder discomfort or pain. Urothelial tissues and cells from affected clinical subjects and asymptomatic controls have been compared for expression of proteins and mRNA. Animal models have also been used to probe urothelial responses to injuries of the urothelium, urethra, or central nervous system, and transgenic techniques are being used to test specific urothelial abnormalities on bladder function. BPS/IC, FIC, and OAB appear to share some common pathophysiology including increased purinergic, TRPV1, and muscarinic signaling, increased urothelial permeability, and aberrant urothelial differentiation. One challenge is to determine which of several abnormally regulated signaling pathways is most important for mediating bladder dysfunction in these syndromes, with a goal of treating these conditions by targeting specific pathophysiology.

## 1. Introduction

Although the cause(s) of urinary bladder functional syndromes is (are) largely unknown, there is an emerging body of evidence to support the involvement of bladder urothelial abnormalities in these illnesses. In this review, we will present published information concerning bladder urothelial abnormalities for two of these syndromes in humans (bladder pain syndrome/interstitial cystitis or BPS/IC, and overactive bladder syndrome or OAB), as well as one that occurs in cats (feline interstitial cystitis or FIC). To aid in understanding the possible relationship of these disorders to each other, we will also summarize those bladder urothelial abnormalities that these disorders have in common, as well as list possible future studies that could help us to understand whether additional similarities may exist for these three syndromes.

## 2. Bladder Urothelial Cell Gene Expression and Function in BPS/IC

BPS/IC is a debilitating chronic painful bladder syndrome that may affect as many as one million people in the USA, 80–90% of whom are women [1, 2]. BPS/IC often has a rapid onset with pain, urgency, and increased frequency of urination and cystoscopic abnormalities including petechial hemorrhages (glomerulations) in approximately 90% of patients or ulcers that extend into the lamina propria (Hunner's ulcers) in approximately 10% [2, 3], though current incidence of Hunner's ulcers is believed to be much lower. While no definite etiology for this syndrome has been identified, bladder urothelial abnormalities are a cardinal finding in biopsies from BPS/IC patients, with the predominant and most consistent histologic findings in the Interstitial Cystitis Data Base Study and other reports including denudation and tears in the bladder urothelium (Hunner's ulcers or glomerulations) [3–5] and/or thinning of the bladder urothelium to 1-2 cell layers thick [5]. Electron microscopic studies confirmed bladder urothelial abnormalities in BPS/IC patients with disruption of the asymmetric unit membrane and bladder urothelial junctions of the surface umbrella cells [6]. The bladder urothelial mucin layer has also been shown to be abnormal in BPS/IC [5], and increased bladder urothelial permeability also has been suggested by some studies [7, 8].

Certain evidence suggests that these findings may result from intrinsic bladder urothelial abnormalities rather than extrinsic damage to the bladder urothelial cells. For example, although tissue from patients with Hunner's ulcers typically contains inflammatory cell infiltrates in the lamina propria (often consisting of T lymphocytes with or without mast cells) [3, 5] little inflammation is usually seen in tissue from the much larger number of patients without ulcers [5], suggesting that inflammation might occur in response to, rather than being a cause of, bladder urothelial damage. In addition, urothelial gene expression also consistently appears to be abnormal in bladder tissue from BPS/IC patients [9–13], with altered levels of specific cell proteins and proteoglycans, providing additional evidence for an intrinsic bladder urothelial cell defect related to an abnormal program of gene expression and/or differentiation. Finally, studies with isolated, explanted bladder urothelial cells from BPS/IC patients have displayed abnormalities consistent with the histopathologic findings, including decreased cell proliferation, abnormal gene expression, and abnormal function as compared to bladder urothelial cell explants from normal controls.

This section will review in more detail published findings regarding each of the bladder urothelial cell abnormalities described in tissue biopsies and/or bladder urothelial cell explants from BPS/IC patients, concentrating on those with initial descriptions of bladder urothelial abnormalities, large studies, and studies in which BPS/IC tissue and cell explants were compared to controls; abnormalities in urine components are beyond the scope of this review. In addition, we will review correlations between urothelial abnormalities in bladder biopsy specimens and abnormalities in explanted bladder urothelial cells when available, as well as published correlations between bladder urothelial abnormalities and the voiding abnormalities found in this illness.

### 2.1. Histopathologic Evidence for Bladder Urothelial Abnormalities in BPS/IC Patients

The earliest published description of histopathologic abnormalities in BPS/IC bladders from patients with Hunner's ulcers described areas where the bladder urothelium was absent and replaced with granulation tissue, as well as underlying increases in inflammatory cells, fibrosis, and capillaries [14]. Since that time, many other studies have also described urothelial abnormalities as one of the predominant histopathologic findings in BPS/IC bladders, including thinning and/or ulceration on the microscopic level in tissue from both patients with Hunner's ulcers as well as nonulcer BPS/IC patients. For example, the first description of electron microscopic findings in bladder tissue from 3 nonulcer BPS/IC patients as compared to findings in tissue from controls with urinary retention and infection or bladder tumors showed consistent evidence for abnormal tight junctions in the BPS/IC but not control groups [15]; this finding was then confirmed by showing significantly greater leakage of radioactive substances across the bladder wall and into the bloodstream following intravesical instillation of either ^24^Na or ^14^C-urea in the BPS/IC patients. However, it appears that only the BPS/IC patients in this study were hydrodistended prior to biopsy. Similar electron microscopic findings were also noted in a subsequent larger study of over 300 BPS/IC patients, in which the bladder tissue had abnormal widening of the space between cells in all layers of the bladder urothelium [16]. Increased absorption of intravesically instilled urea was confirmed in 56 (both nonulcer and ulcer) BPS/IC patients as compared to 31 controls; notably, a subset of the BPS/IC (8 patients) and normal controls (26 patients) that did not undergo hydrodistention under anesthesia prior to urea instillation showed similar results, with 22.1% of the BPS/IC patients having urea absorption versus 4.7% of controls [7]. The same investigators later documented increased pain from intravesical instillation of potassium ions in BPS/IC versus control patients [8], providing additional evidence for abnormal bladder urothelial leakiness in this illness.

Findings from subsequent histologic studies also confirmed the presence of bladder urothelial abnormalities in patients with nonulcer as well as classical (ulcer) BPS/IC. While a comparison of 64 patients with classical BPS/IC to 44 nonulcer patients (including both males and females in both groups) and 20 control women showed evidence for true ulceration only in bladders from the classical BPS/IC group, it also noted that the predominant histopathologic feature in the nonulcer group was multiple mucosal ruptures [3]; apparently with few exceptions both BPS/IC and control biopsies were obtained after hydrodistention. This finding was confirmed in a subsequent larger study by the same group, in which biopsies from nonulcer BPS/IC patients were compared to biopsies from ulcer BPS/IC patients. While 96% of 146 ulcer BPS/IC patients had ulceration, 83% of 64 nonulcer BPS/IC patients were found to have only mucosal ruptures [17]. However, other histopathologic studies, including an earlier study by the same group in which they compared BPS/IC biopsies to controls, have described actual microscopic ulcerations in patients with nonulcer BPS/IC. For example, comparison of 41 nonulcer BPS/IC patients to 16 controls that did not fulfill symptom criteria for BPS/IC revealed primarily mucosal ulceration, as well as degenerative changes to the bladder urothelial cells with vacuolization and detachment in the nonulcer BPS/IC patient group [18]; this study is similarly important for implicating epithelial abnormalities in nonulcer as well as classic BPS/IC because both the nonulcer BPS/IC patients and the controls were hydrodistended prior to biopsy. These latter findings were also described by other groups, including one study that compared bladder tissue from 22 nonulcer BPS/IC patients to tissue from 10 controls (all specimens obtained after hydrodistention) and found evidence for a significantly greater incidence of microscopic mucosal ulceration in nonulcer BPS/IC patients (7 of 22) versus controls (0 of 10) [19]. Another study that compared tissue from 34 BPS/IC patients to tissue from 35 controls also found a significant increase in bladder urothelial denudation in the BPS/IC biopsies. The investigators noted that while 0% of the control specimens had completely denuded epithelium, this finding was noted in approximately 20% of biopsies from BPS/IC patients (with 50% of patients with severe BPS/IC having this finding); however, whether either or both patient groups underwent hydrodistention prior to biopsy is not noted (described in [20]). Finally, the Interstitial Cystitis Database Study by the National Institutes of Health (which studied over 200 both ulcer and nonulcer BPS/IC patients, hydrodistended prior to biopsy) indicated that complete loss of bladder urothelium was highly significantly correlated with nighttime voiding frequency, and the percentage of mucosa denuded of urothelium was highly associated with pain by multivariate predictive modeling [5]. Cluster analysis of the same biopsy specimens indicated that only a small percentage (3.4%) of the BPS/IC patients had complete erosion of the bladder urothelium with cellular inflammation, mastocytosis, and vascular damage, while 96.6% of specimens showed either complete or partial erosion of the bladder urothelium with very limited evidence for any other pathologic features [21].

### 2.2. Evidence for Abnormal Bladder Urothelial Cell Proliferation

Based on these and other reports that (with few exceptions) described an abnormal or absent bladder urothelium in BPS/IC patients, investigators then sought to determine whether these cells had intrinsic abnormalities in their cell proliferation and/or gene expression compared to controls. The first evidence that they may have altered proliferative ability indicated that a subpopulation of basal progenitor cells from BPS/IC patients had an increased proliferative ability as compared to the same basal cells from normal controls when cultured with complete F12 medium containing bovine pituitary extract [22]. However, subsequent studies in which bladder urothelial cells were grown from BPS/IC patients and controls in completely defined cell culture medium (without any growth factors) indicated that bladder urothelial cells explanted from BPS/IC biopsies proliferated at a significantly slower rate than control cells under these conditions [23]. This finding was determined to result from the production of a small Frizzled-8 protein-related glycoprotein (called antiproliferative factor, or APF) that appears to be made uniquely by urothelial cells from BPS/IC (but not control) bladders, and which inhibits human bladder urothelial cell proliferation [24]. This APF was shown to block primarily G2/M in the cell cycle [25, 26] as well as upregulate expression of certain transcription factors associated with decreased cell proliferation (p53 and p21^cip1/waf1^) [26–28].

### 2.3. Evidence for Abnormal Bladder Urothelial Gene Expression

In addition to producing APF, BPS/IC cells have been shown to have abnormal expression or activation of many other gene products as compared to normal controls both in bladder biopsy specimens and in explanted bladder urothelial cells, shown by using a variety of techniques including primarily immunofluorescence staining, Western blotting, polymerase chain reaction, and microarray technology. Unlike the histologic studies described above in which tissue morphology could easily be influenced by a procedure such as hydrodistention (and which therefore generally included hydrodistention of both controls and BPS/IC patients), findings of abnormal protein expression are less likely to be affected during the time between that procedure and tissue fixation; therefore, more of these studies were performed without hydrodistention of the control group(s). Two early papers reported evidence for abnormal HLA antigen expression in BPS/IC bladder urothelial cells, including increased HLA-DR expression both in biopsy specimens as well as in explanted cells (although the latter study used ureteral urothelial tissue and cells for controls and demonstrated findings in cultured cells following treatment with gamma interferon and TNF*α*: unclear whether either group included specimens obtained after hydrodistention) [11, 29]. These reports have been followed by many others that showed significantly abnormal expression of additional urothelial proteins and proteoglycans in bladder biopsies from BPS/IC patients as compared to controls, including* decreases* in chondroitin sulfate proteoglycans (both groups hydrodistended prior to biopsy) [9, 30]; uroplakin (both groups hydrodistended prior to biopsy) [9]; neurokinin receptor 1 (only BPS/IC patients underwent uroflowmetry, postvoid residual and urodynamic investigations) [10]; and the urothelial tight junction proteins zonula occludens 1 [9, 10], junctional adhesion molecule 1, occludin, and claudin 1 [10]. BPS/IC bladder biopsies have also been shown to have* increased *expression of many other bladder urothelial cell genes including nerve growth factor (all control groups also hydrodistended prior to biopsy in [31]; only BPS/IC group hydrodistended prior to biopsy in [32]), E-cadherin (both BPS/IC and control specimens obtained after hydrodistention) [9], inducible nitric oxide synthase (unclear whether some BPS/IC patients and controls did not undergo hydrodistention prior to biopsy) [33, 34], caveolin 1 (no mention of hydrodistention for BPS/IC patients or controls) [35], vascular endothelial growth factor (only BPS/IC patients hydrodistended prior to biopsy) [36], human chorionic gonadotropin beta (no mention of hydrodistention for BPS/IC patients or controls) [37], claudin 2, and a variety of cell signaling receptors including bradykinin B(1) receptor, cannabinoid receptor CB1, muscarinic receptors M3–M5 [10], and transient receptor potential vanilloid 2 (TRPV2) [32]. Increased activated (nuclear) NF*κ*B was also evident in BPS/IC bladder urothelial cells [38]. In addition, qRT-PCR of bladder biopsies showed increased mRNA expression for acid-sensing ion channels (ASIC) 2 and 3, but decreased mRNA expression for transient receptor potential vanilloid 1 (TRPV1) (no mention of hydrodistention for BPS/IC patients or controls) [39]. However, as the biopsies used in the latter two referenced microarray studies [32, 39] included both urothelial and other bladder cells, as protein expression was not evaluated for the same genes, and as functional differences related to ASIC or TRPV expression were not evaluated, the significance of these findings for the bladder urothelium in BPS/IC patients is currently unclear. (Please note that because bladder biopsies from patients with ulcerative BPS/IC may be largely or completely denuded of urothelium even in nonulcerative areas, results from microarray analysis on biopsy specimens exclusively from patients with ulcerative BPS/IC are not included in this review).

Of those gene expression abnormalities identified in BPS/IC tissue specimens, those also noted to be stably abnormally expressed by BPS/IC bladder urothelial cell explants include changes in tight junction and adherens protein expression with decreases in occludin and zonula occludens 1 [40, 41] and claudin 1, 4, and 8 [42], with increases in E-cadherin expression [40, 41]. The decreased tight junction protein expression was also shown to correlate with increased paracellular permeability of radiolabeled tracers* in vitro* [41]. In addition, microarray analysis of BPS/IC cell explants also revealed significant decreases in vimentin, putative tRNA synthetase-like protein, neutral amino acid transporter B, alpha 1 catenin, alpha 2 integrin, and ribosomal protein L27a, and significant increases in arylsulfatase A, phosphoribosyl-pyrophosphate synthetase associated protein 39, and SWI/SNF BAF 170 [40]. The fact that promoters for all these genes with abnormal mRNA expression by microarray analysis have AP-1 and/or Sp-1 binding sites suggests that transcription factors in addition to p53, p21^cip1/waf1^ and NF*κ*B may be abnormal in bladder urothelial cells from BPS/IC patients. In addition to intracellular proteins, BPS/IC cell explants were also shown to secrete increased levels of certain proteins including IL-1, TNF*α*, class I and class II HLA molecules, and certain epithelial cell growth factors (including EGF and IGF1) but decreased levels of other proteins such as HB-EGF [11, 23, 29, 43].

Notably, all abnormalities in gene expression, paracellular permeability, and the secretion of specific epithelial cell growth factors found in BPS/IC cell explants* in vitro* have also been induced in normal bladder urothelial cells by treatment with a synthetic active derivative of APF [24, 40], and the expression of uroplakin and zonula occludens 1 was specifically attenuated by the same APF derivative in a mouse model during bladder urothelial repair [44]. APF was also shown to decrease cell secretion of heparin-binding epithelial growth factor-like growth factor (HB-EGF), as well as an enzyme that cleaves pro-HB-EGF with release of active HB-EGF-matrix metalloproteinase-2 (MMP-2) [28]. Finally, several of these abnormalities (including tight junction protein expression and paracellular permeability) were also normalized in BPS/IC cells by treatment with functional antagonists of APF [45]. Therefore, it is possible that many of the bladder urothelial abnormalities identified in BPS/IC patients result from the production of this toxic factor (APF), and it is also possible that APF antagonists may be helpful for normalizing bladder urothelial gene expression in BPS/IC.

The large number and types of genes whose expression was significantly abnormal in BPS/IC bladder urothelial cells (both in tissue specimens and in culture of explanted cells), along with abnormal levels of specific transcription factors, suggested a pattern of abnormal bladder urothelial cell differentiation in BPS/IC [9, 12, 24, 30]. Additional evidence for abnormal bladder urothelial differentiation in this illness was provided by demonstration of abnormal cytokeratin expression in BPS/IC biopsies (as compared to historical controls), indicating a change from transitional cell to squamous urothelial cytokeratin expression [13]. In addition, in one model to determine the capacity of bladder urothelial cells to differentiate into transitional cells* in vitro*, the absence of a normal differentiation response was noted in cells from 4 of 7 BPS/IC patients [46].

### 2.4. Abnormal Cell Signaling in BPS/IC Bladder Tissue and Explanted Bladder Urothelial Cells

In addition to abnormal expression of those gene products mentioned above, abnormal expression in BPS/IC bladder cells also appears to extend to the production of various cell signaling molecules and signaling receptors, as well as ultimate downstream modification of certain target proteins/enzymes. For example, BPS/IC biopsies displayed increased purinergic (P2X2 and P2X3) receptor protein [47], as did bladder urothelial cell explants (P2X3) following stretch* in vitro* [48]. While the latter study compared cells explanted from biopsies of BPS/IC patients following hydrodistention to those explanted from nonhydrodistended controls, the former study obtained all biopsy specimens from patients and controls at the time of cystectomy or radical prostatectomy (no mention of hydrodistention being performed at the same time). Urothelial biopsies from patients with BPS/IC were also shown to release significantly more ATP in response to stretch or electrical field stimulation than normal bladder urothelial biopsies [49], and BPS/IC bladder urothelial explants showed increased ATP release* in vitro* following either stretch [50] or exposure to exogenous ATP [51] as compared to controls (however, all of these studies used tissue obtained following hydrodistention in BPS/IC patients as compared to nonhydrodistended control tissue). Both P2X3 expression and ATP-stimulated ATP release, which suggested an association between cytokine and purinergic signaling in bladder urothelial cells, could also be induced in control cells by treatment with an active synthetic derivative of APF [52]. In addition, in single cell electrophysiologic studies, explanted IC/PBS bladder urothelial cells displayed reduced inward rectifying potassium current with conductance of the Kir2.1 channel as compared to normal control cells (only BPS/IC patients hydrodistended prior to biopsy) [53]. Finally, BPS/IC explants were also shown to have an enhanced sensitivity to carbachol, suggesting that muscarinic signaling may also be abnormally regulated in BPS/IC (only BPS/IC patients hydrodistended prior to biopsy) [54].

APF has also been shown to affect signaling via certain pathways in bladder urothelial cells, indicating the possibility that these pathways may also be abnormally regulated in BPS/IC cells. For example, HPLC-purified native APF was shown to inhibit Erk/MAPK while stimulating p38MAPK signaling in T24 cells [55], and the latter finding was subsequently corroborated by the finding of increased phospho-p38MAPK in urothelial cells of BPS/IC biopsies as compared to controls (both BPS/IC patients and controls hydrodistended prior to biopsy) [56]. This latter study also demonstrated an increase in phospho-p53 and other proapoptotic proteins in these cells, supporting a possible role for APF in abnormal cell signaling in BPS/IC. In addition, recent studies have shown evidence for inhibition of Wnt/Akt signaling in T24 cells by an active synthetic APF derivative including decreased phosphorylation of Akt, GSK3*β*, and *β*-catenin ser45/thr41, with increased phosphorylation of *β*-catenin ser33,37/thr41 [28]. Quantitative proteomics subsequently confirmed the role of a beta catenin network in HPLC-purified native APF-induced signaling [57]. Whether signaling by APF is mediated directly or indirectly by its known functional receptor CKAP4/p63 [58] is unknown. However, an intriguing recent report indicated that APF induces phosphorylation of CKAP4/p63 ser3, 17, and 19, resulting in nuclear translocation and DNA binding of the APF receptor [59].

Additional evidence for possible abnormal cell signaling in BPS/IC also comes from studies of a similar bladder illness in cats called Feline Interstitial Cystitis, as described in the next section.

### 2.5. Summary

There now exists a large body of data to indicate extensive abnormalities in bladder urothelial cell gene expression, function, and signaling in BPS/IC, with many of these abnormalities being found both in patient tissue specimens and in explanted bladder urothelial cells. These findings have largely been described and/or confirmed in studies in which both patients and controls underwent hydrodistention, indicating that they are not artifactual (i.e., not caused by the procedure alone). The extent and specific types of differences in expression of intracellular and secreted proteins, in components of the various signaling pathways, and in downstream modification of target proteins, are compatible with the hypothesis of aberrant bladder urothelial cell differentiation in this illness. The resultant abnormalities of bladder urothelial cell proliferation, gene expression, and signaling by these abnormally differentiated bladder urothelial cells could ultimately result in bladder urothelial thinning or ulceration, leakiness, and altered downstream afferent neuronal cell activation, causing increased urinary frequency, urgency, and pain which characterizes BPS/IC.

## 3. Urothelial Cell Physiology in Feline Interstitial Cystitis (FIC)

A number of animal models have been used for the study of BPS/IC such as administration of an irritant to otherwise healthy rodents [60]. There is a naturally occurring disease in cats that has been termed feline interstitial cystitis or FIC that shares a number of characteristics of the nonulcerative form of BPS/IC in humans [60, 61].

### 3.1. Alterations in Urothelial Barrier Function

Functional pain syndromes such as BPS/IC have been associated with alterations in the urothelium, which include an alteration or degradation of the barrier function. Similar to patients with BPS/IC, cats diagnosed with FIC exhibit alterations in urothelial ultrastructure and tight junction proteins [62]. Changes in the urothelial barrier can permit water, urea, and noxious substances present in the urine to pass into the underlying tissue (neural and other layers), which may result in symptoms of urgency, frequency, and pain during bladder distension. In addition, disruption of the urothelial barrier may also be due to hormonal and neural mechanisms. Findings of elevated nitric oxide levels have been reported in cats with FIC [63] as well as in patients with BPS/IC, which appear to be similar to those in other epithelia where excess production of NO has been linked to altered epithelial integrity [64].

### 3.2. Alterations in Urothelial Sensory Function

BPS/IC has often been described as a disease of the urothelium, which is likely to communicate in a multidirectional fashion with underlying bladder nerves, smooth muscle, and cells of the immune and inflammatory systems [65]. For example, the increased neural excitability that has been reported in cats with FIC [66, 67] may be affected by changes in release of various transmitters or trophic factors from the urothelium [66]. Augmented release of ATP from the urothelium can lead to painful sensations via sensitization of sensory fibers [68, 69]. The increase in ATP release (evoked by mechanical stimuli) in FIC urothelium [70] is similar to that described in urothelial cells isolated from patients with BPS/IC [71]. In addition, both FIC and BPS/IC patient urothelium exhibited plasticity in purinergic receptor expression [48, 72], which may be linked with changes in sensory pathways. Also, it is hypothesized that physical and emotional stress, via alterations in stress-mediators (such as corticotrophin releasing factor or CRF, as shown in animal models) may exacerbate a number of lower urinary tract disorders including BPS/IC. In FIC urothelium, the functional activity of CRF receptors is altered compared to urothelium from healthy, unaffected cats [73]. Thus, changes in stress-related peptide signaling may result in alterations in urothelial-cell signaling and bladder function. In addition, increased nerve growth factor (NGF) in urine and tissue has been linked with bladder pathologies including idiopathic sensory urgency as well as BPS/IC [74]. Increased NGF in bladder urothelium has been reported in FIC as compared to urothelium from healthy, unaffected cats [75]. Studies have shown that a major source of NGF comes from smooth muscle of the bladder in addition to the urothelium, and altered NGF levels may contribute to altered neural excitability and emergence of bladder pain [76].

### 3.3. Comorbidity in FIC

Patients diagnosed with BPS/IC often exhibit a number of comorbid disorders that can also include other pelvic pain problems (irritable bowel syndrome or fibromyalgia) [77–79]. These individuals typically exhibit diffuse hyperalgesia or allodynia, which suggests the possibility of a generalized dysfunction in pain or sensory processing. Though the etiology is unknown, a number of factors have been proposed that may contribute to or modulate the pathophysiology of such overlapping disorders which can include changes in epithelial sensor/barrier functions, inflammation, or autoimmune involvement [80]. Changes in epithelial barrier or sensory functions are not specific to that of the urinary bladder. For example, patients with gastroesophageal reflux disease (GERD) have been reported to exhibit changes in esophageal epithelial structure and function, similar to that of BPS/IC patients [81]. Thus, similar to BPS/IC, a loss of esophageal epithelial integrity may allow access of luminal acid to underlying afferent terminals that can ultimately lead to esophageal pain. There is evidence that nonneuronal acetylcholine plays a role in sensation, cell-cell signaling, proliferation, and cell growth as well as maintaining barrier function [82]. There is an alteration in expression of components of the nonneuronal acetylcholine machinery in FIC bladder urothelium as well as the esophageal epithelium [83, 84]. It is likely that many of these alterations could contribute to epithelial hypersensitivity and barrier dysfunction that often occur in patients with functional and inflammatory urological as well as gastrointestinal esophageal symptoms.

### 3.4. Summary

There are number of unanswered questions in terms of research that could be done to better relate the animal model to that of the human condition. Use of both animal and human tissue and explants (from different bladder areas) could be used to explore whether similar types of receptors are expressed and to determine whether there are similarities in abnormal signaling between animal and human interstitial cystitis. In addition, a study of sex/gender differences could even influence research on a number of comorbid conditions that share epithelial abnormalities that influence function. These and other research questions could influence treatment paradigms and also increase our understanding of mechanisms underlying urothelial function in health and disease.

## 4. Urothelial Abnormalities in Nonneurogenic, Idiopathic OAB

Overactive bladder (OAB) is a highly prevalent clinical syndrome. The International Continence Society has defined OAB as the presence of urinary urgency, usually accompanied by daytime urinary frequency and nocturia, with or without urgency urinary incontinence,* in the absence of infection or other identifiable etiology* [85]. While there are several possible etiologies for OAB, historically, the mechanism receiving the most attention is increased detrusor smooth muscle (DSM) contractility (overactivity) [86]. This mechanism provides the foundation for using oral antimuscarinics to block DSM overactivity. A newer hypothesis for OAB is augmented bladder urothelial-afferent signaling which gives rise to OAB syndrome and, more generally, lower urinary tract symptoms (LUTS) [87, 88].

Use of animal models to study OAB can be problematic because bladder symptoms cannot be ascertained in an animal and surrogate physiologic and behavioral outcomes have to be utilized. There has been a wide range of animal models used to study OAB, most models involving invasive manipulations or injuries to the bladder (i.e., cyclophosphamide treatment), urethra (i.e., urethral obstruction), neural tissues (i.e., spinal cord injury, autoimmunity to nerves), or pancreas (i.e., creation of diabetes model with streptozotocin). Because, by clinical definition, OAB does not arise secondary to these manipulations or injuries (if LUTS arose from these causes, then the term OAB would not apply), these models are less than ideal. Recently, transgenic animals with alterations specifically targeting the urothelium (urothelial restriction or conditional transgenics) have been developed to test the hypothesis that urothelial dysfunction can drive altered bladder function. Therefore, the contribution of specific urothelial mechanisms can be studied in a “natural”* in vivo* state without need for purposeful manipulations and/or tissue damage. A review of different animal models in OAB research has been published [89]. However, the pathophysiologic relationship of all of these animal models to OAB in humans is unknown.

The more direct approach of searching for evidence of epithelial pathophysiology in the human disease using human OAB urothelial tissue has also been utilized to determine whether urothelial pathophysiology contributes to OAB pathogenesis. However, limited numbers of mechanistic experiments can be performed with human tissues and cells. Perhaps then the best approach to studying urothelial contributions to OAB symptoms is first to identify specific abnormalities from human OAB urothelium and then reproduce these abnormalities in an animal model (e.g., by using an urothelial conditional transgenic approach). If the conditional transgenic animals recapitulate the clinical OAB phenotype, then the urothelial abnormalities can be targeted for clinical treatment (and the transgenic animal can also be used to test different treatments).

The focus of this section is to highlight urothelial research performed using both human OAB urothelium and OAB animal models that do not involve tissue injury. A caveat is that studies on urothelial protein expression in animals often use antibodies which may incur a high degree of nonspecificity when applied to the urothelium [90]. The problems of nonspecific antibody binding (adsorption) (which requires the use of appropriate antibody isotype controls), low antibody sensitivity (which can lead to false negative results), and the presence of nonurothelial cells in the preparations can all lead to spurious results. While some studies have adequately addressed these issues, others have not, and therefore new guidelines have been suggested for future research that include use of multiple techniques to verify protein expression (such as immunolocalization,* in situ* hybridization, knockout transgenics, laser capture microdissection, immunogold electron microscopy, and additional controls). In addition, other emerging techniques such as targeted proteomics using liquid chromatography coupled with tandem mass spectroscopy (LC/MS), that can precisely quantitate proteins without need of antibodies [91, 92], have become available since the time many of the published studies were performed, and their application to urothelial biology remains to be implemented.

### 4.1. Urothelial NGF

The concept of urothelial nerve growth factor (NGF) overexpression (OE) contributing to pathophysiology of OAB is based on the findings from several investigative groups that urine from OAB subjects has increased NGF [93]. It is possible that the increased urinary NGF is from increased secretion by the bladder urothelium, though other tissue types may also be the source of urinary NGF (e.g., nerves, suburothelium, and smooth muscle). A urothelially restricted NGF-OE mouse was therefore created to study what happens to the micturition phenotype [94]. These investigators concluded that the morphological and functional features of the NGF-OE transgenic mice reflected the changes observed in micturition reflex pathways in patients with OAB (and BPS/IC). However, other investigators have found that NGF levels in bladder urothelial tissue from OAB patients were not associated with detrusor overactivity, bladder contractility, or increased bladder sensation [95], suggesting that urothelial NGF may not be the critical regulator of bladder overactivity in OAB. While phases I and II clinical trials of anti-NGF monoclonal antibody (tanezumab) for treatment of pain symptoms of BPS/IC were conducted [96] which showed some benefits, unfortunately, these trials were closed by the FDA because of concerns over avascular hip necrosis from this agent. This agent has not been tested in OAB.

### 4.2. Urothelial Purinergic Signaling

Ferguson first proposed the concept that the bladder urothelium can release ATP in response to stretch, with the released ATP serving as a sensory neurotransmitter [97]. The ATP receptor, P2X3, when constitutively knocked out resulted in a hyporeflexive (hyposensitive) bladder [98]. The role of ATP in OAB has been shown in several studies. Investigators found that urinary ATP was a better predictor than urinary NGF for detrusor overactivity in OAB [99]. Urinary ATP levels in OAB patients decreased after treatment with antimuscarinics [100], and a higher pretreatment urinary ATP level predicted a better response to antimuscarinic therapy. However, the sources of the urinary ATP (e.g., whether from the urothelium, suburothelium, nerves, and/or muscles) were not identified in these studies. When urothelial specimens were obtained surgically from OAB and control subjects and then stretched* in vitro*, investigators found that stretched OAB urothelium released significantly more ATP than control urothelium [101]. This finding suggested that urinary ATP levels in OAB patients could reflect urothelial release. In a fructose-fed animal model of metabolic syndrome, which is associated with OAB [102, 103], P2X3 expression was upregulated in the urothelium and intravesically infused ATP induced greater detrusor overactivity in fructose fed animals compared to controls [104]. This suggested increased urothelial purinergic signaling in the fructose fed animals. Taken together, these data suggest that urothelial purinergic signaling is augmented in OAB.

The role of TRP (transient receptor potential) and muscarinic receptors in modulating ATP release by urothelial cells is discussed below in Section 4.7 on urothelial TRPV1.

### 4.3. Urothelial *β*1-Integrin

Integrins are transmembrane proteins that connect the intracellular cytoskeleton with extracellular matrix allowing cellular sensing of changes in organ shape or changes in force. Investigators theorized that integrins could be upstream regulators of mechanosensory apparatus in the urothelium. This hypothesis was tested by creating a *β*1-integrin urothelially restricted knockout mouse [105]. The micturition behavior phenotype showed increased voiding frequency with small volume voids and evidence of “urinary incontinence” based on urinary spot analyses. Urodynamics also showed increased bladder activity (analogous to detrusor overactivity). Interestingly, the urothelium from this transgenic animal also had a 2-fold increase in urinary ATP concentration compared to wild-type animals. These findings suggest that urothelial integrins are important for regulating bladder mechanosensory transduction, and loss of the *β*1-integrin recapitulates an OAB phenotype.

### 4.4. Urothelial Tight Junction Expression

The quantities of tight junction proteins including zona occludens 1 (ZO-1), occludin, and claudin-4 were shown to be decreased in cultured OAB compared to cultured control urothelial cells (manuscript in preparation). This difference was demonstrated using three complementary techniques: Western blotting, quantitative RT-PCR, and immunofluorescence of cultured cells. There was also increased secretion of matrix metalloproteinase-2 (MMP-2) and HB-EGF into the supernatant by OAB urothelial cells similar to what was detected in BPS/IC urothelial cells [43]. The transcript levels of these three tight junction proteins were increased by inhibiting ornithine decarboxylase (ODC), the rate limiting synthetic enzyme for intracellular polyamine production (see Section 4.5). This suggested that polyamines can modulate the mRNA levels of these tight junction proteins.

### 4.5. Urothelial Polyamines

Using cultured urothelial cells from OAB patients, investigators have found significantly increased intracellular polyamine concentrations of spermine, spermidine, and putrescine in OAB urothelial cells [106, 107]. The increased polyamines, associated with an expected increase in expression of the rate limiting synthetic enzyme, ornithine decarboxylase (ODC), blocked the function (measured electrophysiologically) of the large conductance calcium activated potassium channel (BK). The reason that BK function was measured in this urothelial study [106] was because a previous constitutive BK-knockout mouse was shown to have an OAB phenotype [108]. While there is no question that detrusor smooth muscle from the BK-knockout mice had overactivity, the question raised by the 2009 publication is whether there might also be a urothelial contribution to the OAB phenotype in the constitutive BK-knockout mouse, since BK function is also reduced in the urothelium as well as the detrusor smooth muscle.

The increased polyamines also appeared to affect intracellular calcium rise in response to exogenously added muscarinic agonist, oxotremorine (OXO) [107]. It was shown that OAB urothelial cells reacted (as measured by intracellular calcium rise) more strongly to OXO, compared to control cells, based on dose-response curve. This augmented intracellular calcium reactivity to OXO was abrogated when ODC was blocked, suggesting that polyamine can modulate the intracellular calcium raise in response to muscarinic receptor activation.

### 4.6. Urothelial Muscarinic Signaling

As mentioned above, antimuscarinics are currently the mainstay pharmacologic treatment for OAB. The mechanism of action of these agents is to block overactivity of the detrusor smooth muscle. However, muscarinic blockade might affect urothelial signaling through muscarinic receptors located within the urothelial cells. As presented in the prior section on polyamine signaling, OXO, a muscarinic agonist, caused OAB urothelial cells to have higher maximal response in terms of intracellular calcium rise, compared to control urothelial cells [107]. The half maximal concentration for OXO's effect on intracellular calcium rise was also significantly lower for OAB compared to control urothelial cells. This suggested an increased sensitivity to muscarinic agonists in OAB urothelial cells. Whether this increased calcium response to OXO results in some altered downstream effects was not tested in this paper and remains unknown.

However, one of the downstream effects of urothelial muscarinic receptor activation is ATP release by the urothelial cells. Investigators have studied this mechanism in guinea pig and human urothelium [109]. The human urothelial tissues were obtained from “male patients…undergoing diagnostic endoscopy,” but their clinical symptoms were not described. OXO caused the urothelial tissues in both species to release ATP. The ATP release by the urothelium had a paracrine effect and modulated the underlying detrusor smooth muscle.

Other investigators studied ATP release induced by acetylcholine (ACh) in cultured OAB and control urothelial cells [110]. There was no difference in ATP release between the OAB and control cells in response to ACh, which was surprising as it would be expected that there would be increased ATP release by OAB cells. Possible explanations for this unexpected finding include cultured cells not replicating* in vivo* tissue effects and the low number of OAB subjects studied.

Another downstream effect of urothelial muscarinic receptor activation is release of ACh [111]. These investigators concluded that release of ACh has a negative feedback loop on urothelial ACh release and that the urothelial release of ACh is mediated through mechanisms different than neuronal ACh release (which is vesicular exocytosis). Urothelial ACh release induced by muscarinic receptor activation by OXO was studied in cultured OAB cells [107]. OAB cells released significantly more ACh than control cells, but this was only after 6 hours of relatively high dose (10 *μ*M) OXO exposure. The ACh release was abrogated by inhibition of polyamine synthesis.

Whether urothelial muscarinic signaling plays a central role in regulating bladder function (i.e., contributing to OAB symptoms) is not known at this time. While it has been theorized that antimuscarinic therapies for OAB might target the urothelium, this is speculative at this point without much supportive data. Nevertheless, investigators are continuing to investigate the role of muscarinic receptors on the urothelium.

### 4.7. Urothelial TRPV1

The relevance of TRPV1 (transient receptor potential vanilloid 1) channel in bladder function was studied in the constitutive TRPV1 knockout mouse [114]. This transgenic animal had an OAB bladder phenotype on micturition behavior (significantly higher number of small urine spots/hour) and cystometrogram testing (significantly higher nonvoiding contractions/minute). However, in a different study [115, 116], investigators did not find a difference in frequency of bladder contractions on cystometry between the TRPV1 knockout versus the wild-type animals, though nonvoiding contractions were not calculated. Because this transgenic animal is a constitutive knockout, the contribution of urothelial TRPV1 to bladder functional phenotype is uncertain.

While TRPV1 is typically thought of as expressed in c-fiber bladder afferents, this channel is also found on urothelial cells from both rats [117] and humans [112, 113]. Investigators have studied the role of TRPV1 in OAB by using human urothelial tissues. It was found that TRPV1 mRNA was differentially expressed in the human bladder (different expression levels in trigone versus nontrigone areas). Furthermore, when OAB was separated into “sensory urgency” versus “detrusor overactivity” (two terms which are urodynamic definitions), increased TRPV1 mRNA expression was associated with sensory urgency, but not detrusor overactivity [113].

Cultured OAB urothelial cells exhibited a higher maximal response, as measured by intracellular calcium rise, to capsaicin compared control cells [112]. The dose of capsaicin to elicit a maximal response for both OAB and control cells was 10 *μ*M. There was evidence also for increased TRPV1 channel activity in OAB cells, using electrophysiologic measurements. A result of the increased TRPV1 activity is increased ATP release [110]. However, a recent investigation found that while human bladder urothelium expressed mRNA for TRPV1, cultured human bladder urothelial cells did not respond to capsaicin [118], as measured by intracellular calcium rise, unless a very high dose (10–100 *μ*M) of capsaicin was used.

There is also an interaction between TRPV1 and NGF in bladder function. This was studied in the TRPV1 knockout animal [115, 116]. It was found that NGF-induced bladder overactivity was dependent on TRPV1, as TRPV1 knockout animals did not respond with detrusor overactivity to NGF treatment.

Treatment with resiniferatoxin (RTX), which blocks TRPV1, has been tried for OAB. A single dose (50 nM RTX), placebo-controlled trial was performed in 58 patients with idiopathic detrusor overactivity and urgency incontinence (which would be considered idiopathic OAB) [119]. This trial failed to show a positive benefit of RTX. Another trial, which was open label, using a different subject phenotype including those with neurogenic OAB, found a beneficial effect of 50 nM RTX [120]. It should be noted that trials that showed positive effect for RTX were typically utilizing a neurogenic OAB population (e.g., postspinal cord injury). It appears that the benefits of RTX were more pronounced for neurogenic rather than idiopathic OAB.

The role of urothelial TRPV1 in OAB remains unclear. The controversies with regard to the existence of functional TRPV1 protein within the urothelium was highlighted in a published review [90]. Reasons for differences in findings from the different studies include cell culture techniques which could give rise to differently differentiated cells, presence of nonurothelial cells within cell cultures, lack of sensitivity and specificity of anti-TRPV1 antibodies, and species differences.

### 4.8. Summary

OAB is a clinical syndrome that is defined purely by LUTS in the absence of other disease-defining abnormalities. In order to better understand the contribution of urothelial pathophysiology to LUTS, different approaches have to be utilized. Studies of urothelial biopsies from human subjects provide relevance; however, sufficient amounts of clinical samples are limited and in depth* in vivo* mechanistic studies cannot be performed in humans. Furthermore, the phenotypes of OAB subjects in these translational studies vary greatly (or are not described in detail) thus limiting generalizability. Animal models, specifically urothelially restricted transgenics, are valuable tools to test the hypothesis that perturbations in urothelial function results in altered bladder function, but the relevance of animal models to the human illness must be established. As this field progresses, the research community ought to create animal models justified from findings derived from human OAB urothelial tissues. This would result in creating the most relevant animal models, which then can be used to test treatments targeting the urothelium for OAB. The published literature suggests that urothelial pathophysiology in OAB includes altered purinergic, muscarinic, polyamine, NGF, and TRPV1 signaling and preliminary data shows decreased tight junction protein expression.

## 5. Concluding Remarks

This review highlights investigations that showed urothelial abnormalities in three functional bladder conditions: BPS/IC, FIC, and OAB. A broad sweeping unified, single urothelial etiologic mechanism to explain entirely these 3 conditions is currently not possible given the published data and may not exist. However, the literature suggests that certain types of urothelial aberrations might contribute to the development and/or persistence of LUTS in these three conditions, making it possible that the two functional bladder conditions found in humans (BPS/IC and OAB) may be pathophysiologically related to each other as well as to FIC. A summary of these findings are presented in Table 1. Some of the common themes include altered urothelial differentiation, increased urothelial permeability (due to decreased tight junction proteins), and augmented urothelial “transducer-sensor function” (increased ability of the urothelial cells to release and/or respond to putative neurotransmitters). These urothelial abnormalities may be downstream to a more proximal cause or could be the primary defects. For example, in BPS/IC, an antiproliferative factor (APF) has been identified, completely characterized, synthesized, and shown to induce some of the same abnormalities in expression of cellular and secreted protein expression in both normal urothelial cell explants* in vitro* [24, 26–28, 40–43] and in mouse urothelial cells* in vivo* [44]. APF and/or urothelial growth factors whose expression can be regulated by APF (HB-EGF and EGF) have further been shown to induce certain abnormalities in ATP and potassium channel signaling found in BPS/IC cells [52, 53]. Whether the same or similar factors cause the urothelial abnormalities found in OAB and FIC remains to be determined.

Because the urothelium is comprised of three cell layers (apical, intermediate, and basal) with each layer providing different functional roles, studying the way by which the urothelium might regulate bladder function is complex. However, using mouse genetic tools, investigators are now able to study a primary urothelial defect and how this defect alone, as a proximal cause, can affect bladder function. The findings presented herein support the hypothesis that urothelial abnormalities adversely affect bladder function. A focus on targeting urothelial abnormalities may therefore be very beneficial for advancing treatment outcomes for these functional bladder disorders.

## Figures and Tables

**Table 1:** Evidence for abnormal bladder urothelial cell structure and function in BPS/IC, FIC, and OAB.

Clinical condition	Bladder urothelial abnormality	Source	Reference
BPS/IC	**Structural abnormalities** (e.g., absence of epithelium, mucosal ulceration, mucosal ruptures, abnormal/leaky tight junctions, widening of spaces between cells, cell vacuolization, and urothelial detachment)	Tissue	Hunner 1914 [14]; Eldrup et al. 1983 [15]; Fall et al. 1985 [18]; Said et al. 1989 [16]; Lynes et al. 1990 [19]; Johansson and Fall 1990 [3], Johansson and Fall 1994 [17]; Tomaszewski et al. 2001 [5]; Rosamilia et al. 2003 [20]; Leiby et al. 2007 [21]
	**Abnormal protein expression** (e.g., abnormal cytokeratin expression; decreased chondroitin sulfate proteoglycans, uroplakin, zonula occludens 1, occludin, Junctional adhesion molecule-1, NK1, NK2, TRPV1, ASIC1, and 2; increased HLA-DR, E-cadherin, NFkB, claudin 2, bradykinin B1 receptor, cannabinoid receptor CB1, muscarinic receptors M3-M5, TRPV2, beta-hCG, VEGF, caveolin-1, inducible nitric oxide synthase, ICAM-1, IL1-*α*, and TNF-α)	Tissue	Christmas and Bottazzo 1992 [29]; Liebert et al. 1993 [11]; Hurst et al. 1996 [30]; Abdel-Mageed and Ghoniem 1998 [38]; Slobodov et al. 2004 [9]; Laguna et al. 2006 [13]; Koskela et al. 2008 [33]; Freire et al. 2010 [10], Sánchez-Freire et al. 2011 [39]; Lee and Lee 2011 [36]; Lin et al. 2011 [35]; Schwalenberg et al. 2012 [37]; Logadottir et al. 2013 [34]; Homma et al. 2013 [32]
	**Abnormal protein expression** (e.g., specific production of a frizzled-8-related antiproliferative factor; increased induction of HLD-DR; increased E-cadherin, arylsulfatase A, phosphoribosyl-pyrophosphate synthetase associated protein 39, and SWI/SNF BAF 170; decreased tight junction proteins (including zonula occludens 1, occludin, plus claudins 1, 4, and 8), vimentin, putative tRNA synthetase-like protein, neutral amino acid transporter B, alpha 1 catenin, alpha 2 integrin, and ribosomal protein L27a; decreased secretion of HB-EGF and MMP-2)	Culture	Liebert et al. 1993 [11]; Keay et al. 2003 [23], Keay et al. 2004 [24]; Zhang et al. 2005 [41], Zhang et al. 2007 [42]; Shahjee et al. 2010 [28]
	**Abnormal protein expression** (e.g., decreased uroplakin and zona occludens 1 in animal model with APF)	*In vivo* (*animal model*)	Keay et al. 2012 [44]
	**Functional abnormalities** (e.g., increased intravesical urea absorption)	Tissue	Eldrup et al. 1983 [15]
	**Functional abnormalities** (e.g., increased paracellular permeability)	Culture	Zhang et al. 2005 [41]
	**Functional abnormalities** (e.g., increased intravesical urea absorption, increased sensitivity to intravesical potassium ions)	*In vivo* (*humans*)	Parsons et al. 1991 [7], Parsons et al. 1998 [8]
	**Correlation of urothelial abnormalities with voiding symptoms** (including denudation with nighttime frequency and pain)	Tissue	Tomaszewski et al. 2001 [5]
	**Abnormal cell proliferation**	Culture	Elgavish et al. 1997 [22]; Keay et al. 2003 [23]
	**Abnormal cell signaling** (e.g., Akt and/or beta catenin signaling, increased purinergic signaling, reduced Kir2.1 channel activity, increased intracellular calcium to carbachol, increased ATP release, increased p38MAPK phosphorylation/signaling, and increased p53 phosphorylation)	Culture/Tissue	Sun et al. 2001 [50], Sun et al. 2004 [48]; Tempest et al. 2004 [47]; Sun and Chai 2006 [51]; Kumar et al. 2007 [49]; Gupta et al. 2009 [54]; Shahjee et al. 2010 [28]; Kim et al. 2009 [55]; Yang et al. 2011 [57]; Shie et al. 2012 [56]
	**Physiologic effects of APF** (e.g., G2/M cell cycle block; regulation of transcription factor expression, phosphorylation/palmitoylation/nuclear translocation of CKAP4)	Culture	Abdel-Mageed and Ghoniem 1998 [38]; Rashid et al. 2004 [25]; Kim et al. 2007 [26]; Kim et al. 2012 [27]; Shahjee et al. 2010 [28]; Zacharias et al. 2012 [59]

Feline IC	Increased inducible nitric oxide synthase	Tissue	Birder et al. 2005 [63]
	Urothelial denudation, decreased transepithelial resistance, and increased permeability	Tissue	Lavelle et al. 2000 [62]
	Increased purinergic signaling	Culture	Birder et al. 2003 [70], Birder et al. 2004 [72]

Overactive bladder	Increased purinergic signaling	Tissue	Kumar et al. 2010 [101]
	Increased ornithine decarboxylase	Tissue	Li et al. 2009 [106]
	No difference in NGF (protein ELISA) OAB DO versus OAB non-DO	Tissue	Birder et al. 2007 [95]
	Increased polyamine and block of BK channel	Culture	Li et al. 2013 [107] and Li et al. 2009 [106]
	Increased TRPV1 signaling	Culture	Birder et al. 2013 [110]; Li et al. 2011 [112]; Liu et al. 2007 [113]
	Increased muscarinic signaling (increased intracellular calcium)	Culture	Li et al. 2013 [107]
	Decreased zonula occludens 1, occludin, claudin 4 in cells; decreased secretion of HB-EGF and MMP-2	Culture	Chai et al. 2014 (manuscript in preparation)

Transgenic urothelial restricted models	Decreased β1-integrin Increased NGF	Tissue Tissue	Kanasaki et al. 2013 [105] Schnegelsberg et al. 2010 [94]
